# Supplementary material for: Unzipping haplotypes in diploid and polyploid genomes
Source: Comput Struct Biotechnol J. 2019 Dec 9;18:66–72. doi: 10.1016/j.csbj.2019.11.011 (PMC6938933; doi:10.1016/j.csbj.2019.11.011)
Supplement: Supplementary data 1 [file mmc1.docx]

**Unzipping haplotypes in diploid and polyploid genomes**

Xingtan Zhang^1,2^, Ruoxi Wu^1^, Yibin Wang^1^, Jiaxin Yu^1^, Haibao Tang^1^*

^1^ Center for Genomics and Biotechnology, Fujian Provincial Key Laboratory of Haixia Applied Plant Systems Biology, Key Laboratory of Genetics, Breeding and Multiple Utilization of Corps, Ministry of Education, Fujian Agriculture and Forestry University, Fuzhou, 350002, China.

^2^ State Key Laboratory of Ecological Pest Control for Fujian and Taiwan Crops, College of Plant Protection, Fujian Agriculture and Forestry University, Fuzhou, 350002, China.

* Corresponding author: Haibao Tang, e-mail: [tanghaibao@gmail.com](mailto:tanghaibao@gmail.com)

**Abstract**

Diploid genomes consist of two homologous copies of chromosomes with one from each parent while polyploid genomes contain more than two homologous sets of chromosomes. Most of the reference genome assemblies collapsed haplotypes that represent ‘mosaic’ sequences, ignoring allelic variants that may be involved in important cellular and biological functions. Unzipping haplotypes into distinct sets of sequences has been a growing trend in recent genome studies, as it is an essential tool towards resolving important clinical and biological questions, such as compound heterozygotes, heterosis, and evolution. Herein, we review existing methods for alignment-based and assembly-based haplotype phasing for heterozygous diploid and polyploid genomes, as well as recent advances of experimental approaches for improved genome phasing. We anticipate that full haplotype phasing could become a routine procedure in genome studies in the near future.

**Keywords**

genome assembly; haplotype phasing; ploidy; reference genome; heterozygosity

1. **Introduction**

Assembly of the reference genome is a common route to improve the utilization of the genetic resources for many organisms nowadays. With the availability of single molecule long-read sequencing, such as PacBio SMRT (Single Molecular Real-time) sequencing, Oxford Nanopore Technologies (ONT) and Bionano Genomics, as well as high-throughput chromatin conformation capture (Hi-C) technology, most genome sequencing studies can now be completed to chromosomal level assemblies at a fractional cost compared to just several years ago.

In most reference genome assemblies, typically two homologous copies of every chromosome, one from each parent, were collapsed together and considered as a ‘mosaic’ reference of the two haplotypes in the current diploid genome assembly approaches. Such reference is called a ‘monoploid’ representation which only reflects a single haplotype throughout the genome. For example, the human reference genome (the Genome Reference Consortium, with the most recent release of *GRCh38*) is derived from 13 volunteers living in New York, and only contains the O allele for the ABO blood group locus. Similarly, for polyploid genomes, a single haploid was often targeted in the reference genome, thereby ignoring a large amount of genetic diversity within the sequenced organism.

While the ‘monoploid’ references are much simpler to reason about and compare against, they often fail to capture the diploid or polyploid nature of the organisms and ignore allelic variants that can have potentially important function. Since the initial completion of Human Genome Project (HGP) two decades ago, researchers have been trying to improve the genome assemblies to completely resolve both haplotypes in the same sequencing study. Recent improvements in sequencing technologies and particularly development of long read sequencing provide opportunities to resolve structural variations between haplotypes that are completely absent in the linear reference genome. In human genome studies, reconstruction of both haplotypes is also clinically relevant, for example, when determining the presence of compound heterozygous mutations, as well as for accurate Human Leukocyte antigen (HLA) typing.

Another important application of genome phasing is to study allele-specific expression (ASE) or allelic imbalance (AI), which has been suggested as an important mechanism for causing heterosis. ASE or AI reveals a pattern of preferential expression of one parental allele over another [1]. This differential expression pattern likely results from allelic variants located in *cis*-regulatory elements, which are able to interact with environmental factors to regulate complex expression networks [2] and eventually lead to large phenotypic variation. For instance, evidence increasingly reveals transcriptionally more active alleles with dominant expression patterns are important contributors to heterosis in hybrid rice [3][4]. The current approaches to identify ASE relied on phasing RNA-seq reads onto different haplotypes. Typically, RNA-seq reads produced on Illumina short reads sequencing platforms are aligned to a reference genome and variants that belong to each parental genome are phased and the corresponding reads are further used to quantify the gene expression for their respective alleles [3].

Additionally, identification of allelic variants can also facilitate research of polyploid evolution and inform crop breeding. The complex polyploid sweet potato genome was first assembled into a consensus reference genome and a novel haplotype phasing method successfully generated a haplotype-resolved genome assembly [5]. Phylogenetic analysis using phased allelic variants uncovered a total of six haplotypes, thereby tracking the hexaploidization history in the lineage of sweet potato [5]. In the sugarcane project, we recently developed a novel ALLHiC algorithm that is able to build allele-aware and chromosomal-scale assembly of autopolyploid sugarcane genome by incorporating PacBio long reads sequencing and Hi-C technology [6]^,^[7]. The phased sugarcane genome with individually assembled haplotypes further revealed the complex chromosome rearrangement and evolutionary history in the *Saccharum* lineage [6].

Haplotype phasing has become a fundamental problem in heterozygous and polyploid genome assemblies. Haplotype phasing is the key for the most accurate representation of genetic composition for a given organism. Herein, we review the existing computational approaches for haplotype phasing – including both alignment-based and assembly-based phasing approaches for heterozygous and polyploid genomes. An overview of the two phasing approaches is illustrated in **Figure 1**. We follow up the discussion of computational approaches by a brief summary of experimental advances to assist genome phasing. Finally, the limitation of current approaches as well as future directions are discussed.

1. **Alignment-based haplotype phasing**

Plummeting cost of Next-generation sequencing (NGS) provided large amounts of data to perform genome-wide scale of haplotype phasing and facilitated the continuous development of phasing algorithms during the past two decades. When the reference sequence is available, the most straightforward method of haplotype phasing is by first aligning the whole genome sequencing (WGS) reads, followed by compiling a set of heterozygous genotypes at polymorphic sites and finally pairing the neighboring haplotypes successively (**Figure 1**). The exact pairing information of neighboring sites is provided by the co-occurrence of alleles on the same WGS read or read pair.

Different alignment-based phasing algorithms have been designed based on different optimization criteria or objective functions, including minimum error correction (MEC), Weighted minimum letter flip (WMLF) and Maximum fragment cut (MFC) [8]. These optimization criteria are often NP-hard, and thus often relying on a number of heuristics to speed up the computation. Briefly, MEC aims at reconstruction of the two haplotypes by applying the minimum number of base corrections [9]. Modified from MEC algorithm, WMLF also measures error by the number of base flips [8]. MFC converts the haplotype determination problem to a Max-Cut problem and finds the solution by searching for the maximum distance that can link each SNP edge of a haplotype block. Other algorithms, such as Graph [10], Heuristic dynamic programming [11], Mixture model [12] and Fuzzy conflict graphs [13], are also applicable to genome phasing, each leveraging different statistical models for errors.

A number of haplotype phasing programs based on these algorithms have been reviewed by Je-Keun et., al in 2015 [8]. Therefore, we only focus on the newly developed NGS-based phasing programs since 2015 and highlight their respective strengths as well as limitations (**Table 1**). WhatsHap provides a dynamic programming algorithm to solve the problem of wMEC, which makes the runtime complexity linear to the number of SNPs of reads [14]. HapCUT2, which is an extension of HapCUT program, is able to handle a wide range of sequencing technologies, including NGS short reads, long reads, linked-reads and Hi-C reads. Different from HapCUT, HapCUT2 adopts a likelihood-based model to estimate technology-specific errors and iteratively searches for a subset of variants using max-cut computations in the read-haplotype graph [15, p. 2]. SHAPEIT is a series of software programs that are designed for the estimation of haplotypes based on population-level polymorphism data. The recently developed SHAPEIT3 modified the Markov chain Monte Carlo (MCMC) sampling routine, which speeds up the process and is capable to handle biobank-scale data sets with very low error rates of haplotype switching [16]. As an extension of SHAPEIT3, SHAPEIT4 incorporates a Positional Burrow Wheeler Transform (PBWT) based approach to rapidly select a small set of informative haplotypes from the reference panel [17]. SHAPEIT4 also exhibits sub-linear scaling with sample size and allows for integrating external phasing information such as large reference panels of haplotypes, collections of pre-phased variants and long sequencing reads [17].

**3. Assembly-based haplotype phasing with haploid representation**

In contrast to the alignment-based phasing approaches that are mostly targeted at small variants, assembly-based approaches are often more accurate and can cover larger types of genomic variations, such as large indels and structural variations (**Table 1**). However, heterozygous diploid or polyploid genome assembly can be challenging to assemble due to the presence of multiple haplotypes, leading to ambiguity and redundancies in the initial contig-level assemblies.

To deal with these ambiguities and redundancies, a common practice of assembling heterozygous genomes is to simply reconstruct a single haplotype to represent the whole genome (**Figure 1**). Assembly of heterozygous genomes likely resulted in sequences with high levels of divergence separated in the contig level assembly, i.e. presence of different alleles at the same loci in homologous chromosomes. Due to these common sequences between the haplotypes, the preliminary contig-level assemblies often contain sequences that are over 90% similar between the parental alleles. Pair-wise genome alignment of input contigs can be used to determine redundant contigs, which represent distinct haplotypes from polymorphic regions by comparing amongst the preliminary contigs. This approach has been implemented in Redundans program [23], and successfully assisted heterozygous genome assemblies, such as *Echinochloa crus-galli* [24] and Colorado potato beetle [25].

Another automated pipeline, "HaploMerger" was proposed to reconstruct the allelic relationship of diploid assembly [26]. HaploMerger uses the LASTZ-ChainNet method for whole-genome comparison, and the so-called diploid genome assembly (DGA) diagram is used to describe the relationship between intermediate genes or homologues in the assembly of the diploid genome [26]. HaploMerger showed excellent performance on several polymorphic diploid genomes during its testing and did not introduce new assembly errors, showing its efficacy to analyze and utilize polymorphic genome assembly. HaploMerger2 (HM2) is a major upgrade of the old pipeline, with a re-design of the haploid assembly reconstruction from short and long read diploid assembly [27, p. 2]. The HM2 can handle both low and high heterozygous assemblies, and also provides more flexible assembly error detection and reliable gap closure methods, thereby greatly improving the continuity of the final diploid-level assembly than older pipelines [27, p. 2].

In addition to whole genome alignment approaches, read depth-based methods can also be used to determine allelic contigs in heterozygous diploid genome assembly. For a diploid genome assembly with high level of heterozygosity, a bimodal distribution of read depth are expected which is the basis to classify the contigs of different origins. The “Purge Haplotigs” program is able to utilize read depth of individual contigs to identify contigs that are suspected to be duplicated sequences, which are assumed to be allelic haplotypes that need to be re-assigned in order to obtain a simple, ‘pseudo-haploid’ reference [28]. While relatively simple to derive, it is important to note that this ‘pseudo-haploid’ does not represent a true phasing of the heterozygous genome since the choice of which contigs to retain can still be arbitrary.

**4. Assembly-based haplotype phasing with full haplotype resolution**

While a single haploid representation is relatively straightforward to derive in heterozygous assemblies, it also throws away a large amount of sequence information that belongs to the other haplotypes. Modern sequencing approaches, such as Single-molecule, real-time Sequencing (SMRT) developed by Pacific Biosciences and Oxford Nanopore Technology (ONT) offers long read length, promising overwhelming performance of assembly for complex genomes [18] (**Figure 1**). In particular, long reads have the potential to recover much longer stretches of haplotypes than short reads.

Currently, Pacific Biosciences technology is often used to assemble many plant genomes, but most of the genome assemblies completed thus far are focused on homozygous individuals or inbred lines, yielding a single representative haplotype. However, for many plant crops that are difficult to be inbred as homozygous individuals, such as many tropical fruit crops, the assembly of heterozygous individuals needs to be handled with additional care. In order to solve this problem, several algorithms are developed to simultaneously reconstruct multiple haplotypes during *de novo* genome assembly. Such algorithms seek to go beyond the overly simplistic monoploid representation of the genome, which are especially valuable for heterozygous diploid and polyploid organisms.

The FALCON and FALCON-Unzip algorithm provide a clean solution that enables sequential assembly of the original sequencing data and ongoing identification of the phased diploid genome [19]. Firstly, FALCON reads sequence alignment from corrected PacBio sequence data, and then builds a string graph based on read overlaps [20]. During this process, the string graph typically contains multiple groups of "haplotype fusion" which are overlapping read groups that show up as ‘bubbles’ in the graph. The ‘bubbles’ represent major structural variations and highly divergent regions between homologous sequences. To resolve these ‘bubbles’ within the assembly graph, FALCON-Unzip analyzes haplotype fusion groups and finds hybrid variants as a basis to ‘unzip’ the otherwise fused haplotypes. One haplotype, often arbitrary, is identified first as a primary path in the graph, or ‘primary’ contigs; while the other haplotype, representing an alternative path, is called ‘associated’ contigs. This method was used to re-assemble the F1 genome from crossing Columbia-0 (col-0) and Cape Verde Island-0 (Cvi-0) ecotypes of *Arabidopsis thaliana*, *Vitis vinifera CV* (*Cabernet Sauvignon*) and highly heterozygous wild diploid *Clavicorona pyxidate* [19]. These haplotype-resolved assemblies reflect a more realistic representation of their respective genome and allow the study of haplotype structure and heterozygosity in much better accuracy and resolution.

A more recent approach, “trio binning” is proposed to simplify haplotype assembly by addressing allelic variation prior to assembly [21], which has been implemented in the CANU assembler [22]. Compared with existing methods, the effectiveness of this method increases with the level of heterozygosity. Trio binning starts by partitioning the long reads from the offspring into haplotype specific groups, guided by genome sequencing in each of the two parents. After the partition, each haploid is then assembled independently, resulting in complete diploid reconstruction. To illustrate the utility of “trio binning”, F1 hybrid between cattle subspecies *Bos taurus taurus* and *Bos taurus indicus* were sequenced and fully resolved into two parent haplotypes, with a NG50 of haplotig size of over 20 Mb with 99.998% accuracy, which exceeds the quality of current bovine reference genome [21]. While highly accurate, the setup of “trio binning” requires the parent to be known *a priori* as well as sequenced in order the phase the F1 genome. This requirement not only results in higher cost, but also can be limiting in the scenarios such as for the plant species are either collected from the wild or has less developed breeding programs so that the parentage information may not be available.

**5. Chromosomal scale of haplotype reconstruction using Hi-C technology**

Genome assembly incorporating PacBio long reads sequencing and proximity ligation-based methods is an efficient approach to construct chromosomal level genome assembly. High-throughput chromatin conformation capture (Hi-C) is a technology derived from chromatin conformation capture (3C) technology, which combines chromatin proximity ligation method and high-throughput sequencing technology to obtain a fine map of chromatin interaction across the whole chromosome [29]. Cross-linked chromatin is cleaved by restriction enzymes and proximity ligated *in situ* to obtain interacting DNA fragments. The ligated DNA fragments are captured by biotin and then sequenced by paired-end sequencing. The interacting DNA fragments, shown as pairs of linked reads, reveal long-distance information about the grouping and linear organization of sequences across the entire chromosomes [30]. The probability of intrachromosomal contacts decays rapidly with linear distance following a power law, but still interacts with a much higher probability with loci on the same chromosome (intra-chromosomal) than loci on different chromosomes (inter-chromosomal), with possible interaction even when separated by over 200 Mb on the same chromosome [31]. Based on the proximity linkage information, Hi-C data can effectively identify linkage between contigs or scaffolds, allowing contigs being linked to nearly entire chromosome-scale.

In the past decade, Hi-C based assembly approaches have become broadly available to generate reliable chromosome-scale *de novo* assemblies of the genome projects in mammals, plants and insects. Hi-C data can also be used to phase genome onto separate haplotypes at chromosome-scale since homologous chromosomes occupy distinct territories in nuclei [32], which could be used to distinguish different haplotypes. Therefore, based on these distinct DNA structural domains, haplotype-aware chromosome-level assemblies using Hi-C based methods have been published and successfully applied on several complex polyploid genomes recently, such as the bread wheat [33], peanut [34] and cotton [35] genomes.

One recently published genome phasing and scaffolding software is FALCON-Phase developed by Phase Genomics [36]. FALCON-Phase is capable of phasing contigs or scaffolds onto high-quality haplotype genome assemblies by integrating long-read sequencing data and Hi-C chromatin contact data of a diploid individual [36]. The pipeline builds upon the aforementioned FALCON-Unzip, which generates phased blocks with reduced haplotype switch errors. FALCON-Phase can address the problem of switches and backfill homozygous regions to produce chromosome-level phased diploid genome assemblies.

However, FALCON-Phase pipeline is designed for phasing diploid genomes and does not yet support the construction of chromosomes for polyploid genomes. To address this limitation, a new pipeline of Hi-C scaffolding named ALLHiC was specifically developed for polyploid genomes [7]. ALLHiC uses a novel pruning step to remove the Hi-C links that are allelic or cross-allelic (linkage between different haplotypes). Such links are typically problematic during phasing since they often prevent the haplotypes from getting partitioned separately. Consequently, ALLHiC enables the accurate phasing of allopolyploid and heterozygous diploid genomes to construct chromosome-scale haplotype assemblies by combining ultra-long-range haplotype information in Hi-C data with a high-quality draft assembly. It has been applied successfully to two chromosome-scale autopolyploid genomes, including an auto-tetraploid [6] and an auto-octoploid sugarcane genomes [7]. In addition to autopolyploid genomes, ALLHiC is applicable to a wide range of genomes including simple diploid, heterozygous and allopolyploid genomes.

**6. Experimental methods targeted at haplotype-resolved genome sequencing**

The initial Human Genome Project was primarily carried out through the hierarchical sequencing of large-insert clones with long DNA fragments (50-200 kb) inserted in bacterial artificial chromosomes (BACs) [37]. Similarly, fosmids can also be used as DNA vectors as it utilizes F-plasmid origin of replication and partitioning mechanisms to allow cloning of large DNA fragments. Sequencing of fosmid libraries has been successfully applied to highly heterozygous genomes, such as the heterozygous diamondback moth genome [38]. Fosmid and BAC clones represent a ‘divide-and-conquer’ approach, by breaking the genome into chunks that are much easier to assemble but still retaining the haplotype information since each individual clone represents a single haplotype.

Linked-Reads, a new sequencing technology developed by 10x Genomics, holds the promise to whole genome haplotype phasing at a relatively low cost. This technology leverages microfluidics to partition and barcode high molecular weight DNA molecules, which are then subject to Illumina short-reads sequencing. Paired reads that contain the same barcode are considered to be derived from the same haplotype and linked together during the genome assembly process, which greatly reduces the complexity of genome assembly. Using the Linked-Reads technology, a recent research study on human genomes reveals a comprehensive list of alternative haplotypes in diverse populations [39].

Another related technology, single-tube long fragment read (stLFR) technology developed by Beijing Genomic Institute (BGI), recently showed its potential to haplotyping and *de novo* assembly [40]. Construction of stLFR libraries involves the integration of transposons into long fragments of DNA molecules, which are further mixed with beads containing shared adapters. After the genomic DNA is captured onto the beads, the transposons are ligated to the barcode adapters and then the co-barcoded DNA fragments are subjected to paired end reads sequencing [40].

Clones, linked reads and long fragment reads all provide long-range linkage experimental evidences at range of hundreds of kilobases to several megabases in length in order to support the phasing of haplotypes – extending well beyond the range that are typically offered by a single WGS read including the latest nanopore reads. Both alignment-based and assembly-based approaches are capable of utilizing these experimental evidences during genome phasing. The popularity of the experimental approaches for phasing would be largely determined by their respective throughput and cost.

**7. Summary and Outlook**

Even though there are a number of existing approaches, both computational as well as experimental, it is still challenging to achieve complete and fully accurate haplotype phasing. Currently, there are two overall strategies for haplotype phasing that we reviewed (**Figure 1**). The first class of haplotype phasing methods relies on variant identification from whole genome sequencing reads. The heterozygous variants in a single diploid or polyploid genome are classified into the same block if they are present in the same read or linked read pair. We refer to this class of phasing strategies as alignment-based haplotype phasing, which only reports phased allelic variants that are typically small variants, such as SNPs or small indels. Since reads from larger variants are often missed in the genome alignments, the alignment-based phasing method is not able to recover larger indels or structural variations, such as the human HLA loci or the plant *S*-locus that are known to be structurally diverse. The second class of haplotype phasing is assembly-based haplotype construction. For this strategy, full-length sequences for individual haplotypes are produced, which are often the preferred phasing method when available due to the higher resolution provided than the alignment-based phasing.

Currently, most state-of-the-art algorithms employ the alignment-based strategy to determine adjacent loci based on Illumina short reads and/or PacBio long reads [8], such as HAPCUT2 [9], WHATSHAP [14] and WinHAP2 [42]. However, phasing with Illumina short reads often produce limited length of phased blocks since the distance of adjacent polymorphic sites can exceed the length of a typical Illumina read. Although long reads sequencing technology is able to extend haplotype blocks in some cases, it suffers from lots of sequencing errors, leading to high level of switch errors that mix allelic information in the same block.

For the assembly-based strategy, newer generation of genome assemblers such as FALCON-Phase and ALLHiC, are able to separate allelic contigs and produce chromosomal scale of genome assemblies for heterozygous diploid or polyploid genomes [12][7]. However, with the current implementation, we still have a high level of chimeric contig assembly or collapsed sequences that could lead to a large proportion of mis-joined scaffolds or even result in missing chromosomes [7]. At present, neither of the two strategies are perfect and both can cause significant portion of errors during the reconstruction of haplotypes.

Further developments on haplotype phasing and haplotype-resolved genome assembly techniques are needed [43]. Lately, Pacific Biosciences launches the new Sequel II system with much higher throughput than the previous Sequel system. This new system is able to deliver highly accurate individual long reads (HiFi reads), taking advantage of long-read technology with much improved accuracy (over 99.9% accuracy). *De novo* assembly of a human genome using HiFi reads produced a contiguous and accurate genome with a contig N50 of over 15 Mb and consensus accuracy of 99.997% [44]. With the advent of similar new sequencing technologies as well as accompanying advances in genome assembly algorithms, full haplotype resolution of heterozygous genomes and complex polyploid genomes may soon become a routine procedure in genome studies.

**Acknowledgements**

This work was supported by the National Key Research and Development Program of China (No. 2016YFD0100305 to H.T.), a National Natural Science Foundation of China grant (No. 31701874 to X.Z.), a Natural Science Found Foundation of Fujian grant (No. 2018J01604 to X.Z.) and State Key Laboratory of Ecological Pest Control for Fujian and Taiwan Crops (No. SKL2018001 to X.Z.).

**Figure legend**

**Figure 1. Overview of the two main classes of haplotype phasing strategies.** The left panel is alignment-based and the right one is assembly-based haplotype phasing workflow, respectively. In the alignment-based haplotype phasing, reads are sequenced with relatively low coverage (<30x) and are mapped to a reference genome for variant calling. Afterwards, linked variants are extended into phased blocks each containing a number of neighboring SNPs represented as 0(REF)/1(ALT). In the assembly-based haplotype phasing, much deeper sequencing is typically carried out using a variety of sequencing technologies. Allele-aware *de novo* assembly can be achieved using Falcon-unzip or Canu trio-binning methods. When working with multiple haplotypes, primary contigs can be selected as an arbitrary haplotype representation, e.g. using purge_haplotigs, for downstream analysis. Alternatively, full set of haplotypes can be resolved through Hi-C technology, e.g. using ALLHiC.

**References**

[1] U. Gaur, K. Li, S. Mei, and G. Liu, “Research progress in allele-specific expression and its regulatory mechanisms,” *J. Appl. Genet.*, vol. 54, no. 3, pp. 271–283, Aug. 2013.

[2] D. A. Knowles *et al.*, “Allele-specific expression reveals interactions between genetic variation and environment,” *Nat. Methods*, vol. 14, no. 7, pp. 699–702, Jul. 2017.

[3] X. Huang *et al.*, “Genomic analysis of hybrid rice varieties reveals numerous superior alleles that contribute to heterosis,” *Nat. Commun.*, vol. 6, no. 1, p. 6258, May 2015.

[4] L. Shao *et al.*, “Patterns of genome-wide allele-specific expression in hybrid rice and the implications on the genetic basis of heterosis,” *Proc. Natl. Acad. Sci.*, vol. 116, no. 12, pp. 5653–5658, Mar. 2019.

[5] J. Yang *et al.*, “Haplotype-resolved sweet potato genome traces back its hexaploidization history,” *Nat. Plants*, vol. 3, no. 9, pp. 696–703, Sep. 2017.

[6] J. Zhang *et al.*, “Allele-defined genome of the autopolyploid sugarcane Saccharum spontaneum L.,” *Nat. Genet.*, vol. 50, no. 11, pp. 1565–1573, Nov. 2018.

[7] X. Zhang, S. Zhang, Q. Zhao, R. Ming, and H. Tang, “Assembly of allele-aware, chromosomal-scale autopolyploid genomes based on Hi-C data,” *Nat. Plants*, vol. 5, no. 8, pp. 833–845, Aug. 2019.

[8] J.-K. Rhee, H. Li, J.-G. Joung, K.-B. Hwang, B.-T. Zhang, and S.-Y. Shin, “Survey of computational haplotype determination methods for single individual,” *Genes Genomics*, vol. 38, no. 1, pp. 1–12, Jan. 2016.

[9] P. Bonizzoni, R. Dondi, G. W. Klau, Y. Pirola, N. Pisanti, and S. Zaccaria, “On the Minimum Error Correction Problem for Haplotype Assembly in Diploid and Polyploid Genomes,” *J. Comput. Biol.*, vol. 23, no. 9, pp. 718–736, Sep. 2016.

[10] J. Duitama, T. Huebsch, G. McEwen, E.-K. Suk, and M. R. Hoehe, “ReFHap: a reliable and fast algorithm for single individual haplotyping,” in *Proceedings of the First ACM International Conference on Bioinformatics and Computational Biology - BCB ’10*, Niagara Falls, New York, 2010, p. 160.

[11] M. Xie, J. Wang, and T. Jiang, “A fast and accurate algorithm for single individual haplotyping,” *BMC Syst. Biol.*, vol. 6, no. Suppl 2, p. S8, 2012.

[12] H. Matsumoto and H. Kiryu, “MixSIH: a mixture model for single individual haplotyping,” *BMC Genomics*, vol. 14, no. S2, p. S5, Feb. 2013.

[13] S. Mazrouee and W. Wang, “FastHap: fast and accurate single individual haplotype reconstruction using fuzzy conflict graphs,” *Bioinformatics*, vol. 30, no. 17, pp. i371–i378, Sep. 2014.

[14] M. Patterson *et al.*, “WHATSHAP: Weighted Haplotype Assembly for Future-Generation Sequencing Reads,” *J. Comput. Biol.*, vol. 22, no. 6, pp. 498–509, Jun. 2015.

[15] P. Edge, V. Bafna, and V. Bansal, “HapCUT2: robust and accurate haplotype assembly for diverse sequencing technologies,” *Genome Res.*, vol. 27, no. 5, pp. 801–812, May 2017.

[16] J. O’Connell *et al.*, “Haplotype estimation for biobank-scale data sets,” *Nat. Genet.*, vol. 48, no. 7, pp. 817–820, Jul. 2016.

[17] O. Delaneau, J.-F. Zagury, M. R. Robinson, J. Marchini, and E. Dermitzakis, “Integrative haplotype estimation with sub-linear complexity,” Bioinformatics, preprint, Dec. 2018.

[18] A. Rhoads and K. F. Au, “PacBio Sequencing and Its Applications,” *Genomics Proteomics Bioinformatics*, vol. 13, no. 5, pp. 278–289, Oct. 2015.

[19] C.-S. Chin *et al.*, “Phased diploid genome assembly with single-molecule real-time sequencing,” *Nat. Methods*, vol. 13, no. 12, pp. 1050–1054, Dec. 2016.

[20] E. W. Myers, “The fragment assembly string graph,” *Bioinformatics*, vol. 21, no. Suppl 2, pp. ii79–ii85, Sep. 2005.

[21] S. Koren *et al.*, “De novo assembly of haplotype-resolved genomes with trio binning,” *Nat. Biotechnol.*, vol. 36, no. 12, pp. 1174–1182, Dec. 2018.

[22] S. Koren, B. P. Walenz, K. Berlin, J. R. Miller, N. H. Bergman, and A. M. Phillippy, “Canu: scalable and accurate long-read assembly via adaptive *k* -mer weighting and repeat separation,” *Genome Res.*, vol. 27, no. 5, pp. 722–736, May 2017.

[23] L. P. Pryszcz and T. Gabaldón, “Redundans: an assembly pipeline for highly heterozygous genomes,” *Nucleic Acids Res.*, vol. 44, no. 12, pp. e113–e113, Jul. 2016.

[24] L. Guo *et al.*, “Echinochloa crus-galli genome analysis provides insight into its adaptation and invasiveness as a weed,” *Nat. Commun.*, vol. 8, no. 1, p. 1031, Dec. 2017.

[25] S. D. Schoville *et al.*, “A model species for agricultural pest genomics: the genome of the Colorado potato beetle, Leptinotarsa decemlineata (Coleoptera: Chrysomelidae),” *Sci. Rep.*, vol. 8, no. 1, p. 1931, Dec. 2018.

[26] S. Huang *et al.*, “HaploMerger: Reconstructing allelic relationships for polymorphic diploid genome assemblies,” *Genome Res.*, vol. 22, no. 8, pp. 1581–1588, Aug. 2012.

[27] S. Huang, M. Kang, and A. Xu, “HaploMerger2: rebuilding both haploid sub-assemblies from high-heterozygosity diploid genome assembly,” *Bioinformatics*, vol. 33, no. 16, pp. 2577–2579, Aug. 2017.

[28] M. J. Roach, S. A. Schmidt, and A. R. Borneman, “Purge Haplotigs: allelic contig reassignment for third-gen diploid genome assemblies,” *BMC Bioinformatics*, vol. 19, no. 1, p. 460, Dec. 2018.

[29] E. Lieberman-Aiden *et al.*, “Comprehensive Mapping of Long-Range Interactions Reveals Folding Principles of the Human Genome,” *Science*, vol. 326, no. 5950, pp. 289–293, Oct. 2009.

[30] N. L. van Berkum *et al.*, “Hi-C: A Method to Study the Three-dimensional Architecture of Genomes.,” *J. Vis. Exp.*, no. 39, p. 1869, May 2010.

[31] A. D. Schmitt, M. Hu, and B. Ren, “Genome-wide mapping and analysis of chromosome architecture,” *Nat. Rev. Mol. Cell Biol.*, vol. 17, no. 12, pp. 743–755, Dec. 2016.

[32] J. O. Korbel and C. Lee, “Genome assembly and haplotyping with Hi-C,” *Nat. Biotechnol.*, vol. 31, no. 12, pp. 1099–1101, Dec. 2013.

[33] M. Maccaferri *et al.*, “Durum wheat genome highlights past domestication signatures and future improvement targets,” *Nat. Genet.*, vol. 51, no. 5, pp. 885–895, May 2019.

[34] W. Zhuang *et al.*, “The genome of cultivated peanut provides insight into legume karyotypes, polyploid evolution and crop domestication,” *Nat. Genet.*, vol. 51, no. 5, pp. 865–876, May 2019.

[35] Y. Hu *et al.*, “Gossypium barbadense and Gossypium hirsutum genomes provide insights into the origin and evolution of allotetraploid cotton,” *Nat. Genet.*, vol. 51, no. 4, pp. 739–748, Apr. 2019.

[36] Z. N. Kronenberg *et al.*, “Extended haplotype phasing of *de novo* genome assemblies with FALCON-Phase,” Genomics, preprint, May 2018.

[37] International Human Genome Sequencing Consortium, “Initial sequencing and analysis of the human genome,” *Nature*, vol. 409, no. 6822, pp. 860–921, Feb. 2001.

[38] M. You *et al.*, “A heterozygous moth genome provides insights into herbivory and detoxification,” *Nat. Genet.*, vol. 45, no. 2, pp. 220–225, Feb. 2013.

[39] K. H. Y. Wong, M. Levy-Sakin, and P.-Y. Kwok, “De novo human genome assemblies reveal spectrum of alternative haplotypes in diverse populations,” *Nat. Commun.*, vol. 9, no. 1, p. 3040, Dec. 2018.

[40] O. Wang *et al.*, “Efficient and unique cobarcoding of second-generation sequencing reads from long DNA molecules enabling cost-effective and accurate sequencing, haplotyping, and de novo assembly,” *Genome Res.*, vol. 29, no. 5, pp. 798–808, May 2019.

[41] P. Edge, V. Bafna, and V. Bansal, “HapCUT2: robust and accurate haplotype assembly for diverse sequencing technologies,” *Genome Res.*, vol. 27, no. 5, pp. 801–812, May 2017.

[42] W. Pan, Y. Zhao, Y. Xu, and F. Zhou, “WinHAP2: an extremely fast haplotype phasing program for long genotype sequences,” *BMC Bioinformatics*, vol. 15, no. 1, p. 164, 2014.

[43] M. C. Schatz and A. Cosgrove, “Graph genomes article collection,” *Genome Biol.*, vol. 20, no. 1, pp. 25, s13059-019-1636–0, Dec. 2019.

[44] A. M. Wenger *et al.*, “Accurate circular consensus long-read sequencing improves variant detection and assembly of a human genome,” *Nat. Biotechnol.*, Aug. 2019.
